# Modulating arm swing via haptic cueing alters interlimb neural coupling in older adults

**DOI:** 10.3389/fphys.2025.1657092

**Published:** 2025-09-22

**Authors:** Ines Khiyara, Ben Sidaway, Babak Hejrati

**Affiliations:** ^1^ Biorobotics and Biomechanics Lab, Department of Mechanical Engineering, University of Maine, Orono, ME, United States; ^2^ School of Physical Therapy, Husson University, Bangor, ME, United States

**Keywords:** coordination, interlimb, neural coupling, intermuscular coherence, aging, haptic cueing, arm swing, wearable system

## Abstract

**Purpose:**

Age-related gait impairments are strongly associated with increased fall risk, disability, and mortality. While traditional rehabilitation focuses on the lower limbs, arm movements play a key role in stabilizing gait through interlimb neural coupling. This study investigates whether rhythmic haptic cueing of arm swing, which enhances gait, affects interlimb neuromuscular coordination in older adults.

**Methods:**

Seventeen older adults (mean age = 73.2 ± 6.0 years) completed three walking conditions: Baseline walking, self-selected Fast walking, and walking while rhythmically receiving haptic cues (Cueing) to increase arm swing frequency and walking speed. Gait speed, arm range of motion (ROM), and intermuscular coherence were analyzed using inertial measurement units (IMUs) and surface Electromyography (sEMG). Coherence and directionality analyses were performed in the alpha (8–15 Hz), beta (15–30 Hz), and gamma (30–60 Hz) frequency bands to quantify neural coupling and intermuscular directionality.

**Results:**

Rhythmic Cueing significantly increased arm ROM and gait speed compared to Baseline walking, with improvements comparable to Fast walking. Overall upper–lower limb coherence increased in the alpha and beta bands during Cueing compared to Baseline, with Cueing also exceeding Fast in the alpha band. In specific muscle pairings, significant alpha-band effects were observed in contralateral shoulder–leg pairs, specifically between the left anterior deltoid and right rectus femoris, and between the left posterior deltoid and right biceps femoris. Directionality analysis revealed dominant zero-lag coherence, reflecting shared subcortical and cortical drive in the alpha and beta/gamma bands, respectively, and greater forward-lag coherence during Cueing compared to Baseline, indicating enhanced cortical arm-to-leg influence.

**Significance:**

These findings demonstrate that externally cued arm swing can modulate gait performance and potentially interlimb neural coupling, activating both subcortical and cortical pathways. Rhythmic haptic cueing shows promise as an intervention for older adults, supporting its potential integration into home-based gait rehabilitation programs.

## 1 Introduction

Gait impairments are a significant concern in the aging population, as slow or unstable walking is strongly associated with an increased risk of falls, disability, and even mortality (Nonnekes et al., 2025; Montero-Odasso et al., 2022; Verghese et al., 2006). Recent studies confirm that even small declines in gait speed or stability predict higher rates of hospitalization and loss of independence (Nonnekes et al., 2025; Montero-Odasso et al., 2022; Hardy et al., 2007). A combination of musculoskeletal, sensory, and neural changes causes these impairments. Age-related neurodegeneration, including early changes that precede Alzheimer’s disease, is now recognized as a key contributor to gait decline even in the absence of overt cognitive symptoms (Ali et al., 2025; Allali et al., 2016). Age-related reductions in interlimb coordination and balance are well documented, with older adults exhibiting greater instability and slower recovery after perturbations, reflecting diminished neural control and adaptability (Krasovsky et al., 2012; Krasovsky et al., 2014; Bruijn et al., 2010; Van Hoornweder et al., 2022; Rezaei et al., 2024; Noghani et al., 2025).

Traditional rehabilitation approaches primarily target the legs, but given the growing evidence that arm movements play a significant role in walking, integrating arm swing into gait rehabilitation warrants further investigation. Arm swing is not merely a passive result of trunk rotation; rather, it actively contributes to rhythm regulation, energy conservation, and body stabilization during gait (Meyns et al., 2013; Punt et al., 2015; Bruijn et al., 2010). Restricting arm swing has been shown to increase instability (Bloom and Hejrati, 2021), whereas enhancing it can improve gait stability and coordination, potentially reducing the risk of falls (Krasovsky et al., 2012; Lamontagne and Fung, 2004; Bruijn et al., 2010; Thompson et al., 2017).

Recent work shows that training interventions such as wearable devices can be used to drive arm swing with rhythmic haptic cues (Noghani et al., 2023). Our previous study using a wearable haptic cueing system to shorten arm swing cycle time (CT) by 20%, led to a 30.2% increase in arm range of motion (ROM) and an 18.2% increase in walking speed in older adults (Khiyara et al., 2025). These results suggest that cueing the arms can improve gait speed, symmetry, and perceived balance and coordination in older adults.

While these findings demonstrate the potential for external cueing to enhance arm swing and overall gait performance, the underlying neural mechanisms that mediate these improvements remain unclear. In particular, it is not yet known whether changes in arm movement directly influence leg motion through neural pathways or if both are modulated by a shared central drive. To investigate this, electromyography (EMG) can be used to explore the directionality and coordination of neural signals between the limbs during locomotion.

To better understand these potential neural pathways, directional coherence analysis of EMG signals offers a valuable tool. This technique not only assesses the frequency of muscle activation patterns but also identifies the directionality of neural influence between limbs. Specifically, coherence can be decomposed into three components: zero-lag coherence, indicating shared input; forward-lag coherence, suggesting arm-to-leg influence; and reverse-lag coherence, indicating leg-to-arm influence (Halliday, 2015; Weersink et al., 2021a). This partitioning allows researchers to determine whether limb coordination arises from a common neural source or reflects directional drive between arms and legs. For example, Weersink et al. (2021a) demonstrated that during gait, forward-lag coherence from arm to leg muscles reflects top-down cortical influence, suggesting a potential mechanism by which arm movement could modulate leg activity during walking.

While our previous study (Khiyara et al., 2025) demonstrated the benefits of arm swing training in older adults in terms of key spatiotemporal gait parameters, the current study aims to investigate the underlying neural mechanisms that may explain these improvements. Prior work has shown that arm swing is neurally coupled with leg movement during walking, involving shared cortical and subcortical control pathways, and that gait-related arm swing can drive lower limb muscles, as demonstrated by significant alpha and beta/gamma intermuscular coherence between upper and lower limbs (Weersink et al., 2021a). Alpha-band coherence, in particular, is often linked to subcortical rhythmic control and may be enhanced through synchronized arm swing driven by rhythmic cueing. Moreover, challenging walking tasks have been reported to increase intermuscular coherence (Da Silva Costa et al., 2024; Hüche Larsen et al., 2024). For instance, beta-band intermuscular coherence increased during more complex balance tasks such as beam walking (da Silva Costa et al., 2024; De Freitas et al., 2025). Similarly, proprioceptively challenging or proactive locomotor conditions are associated with increased EMG-EMG coherence, indicating augmented functional coupling under heightened task demands (De Freitas et al., 2025; Da Silva Costa et al., 2024). In addition, Kerkman et al. (2020) found that intermuscular coherence in the 4–22 Hz range increases during 1:1 arm–leg coordination (Kerkman et al., 2020), a pattern associated with faster walking speeds. While increased task difficulty has been associated with elevated intermuscular coherence, the specific effect of walking speed alone remains unclear. Faster walking in older adults may itself represent a more demanding task, potentially recruiting additional neural resources that support enhanced interlimb coupling.

We therefore propose that a wearable haptic cueing system which can increase arm swing rhythm and amplitude, as well as promote faster walking may influence interlimb neural coordination, potentially enhancing intermuscular coherence, particularly in the alpha (8–15 Hz) and beta (15–30 Hz) frequency bands. We hypothesize that both rhythmic cueing and fast walking can enhance intermuscular coherence and forward-lag coherence (arm → leg), indicating top-down neural drive. This study leverages advanced signal processing and directional coherence analysis to determine whether gait improvements arise from strengthened subcortical pathways, increased cortical drive, or more efficient bidirectional coupling between the limbs. These findings will guide the development of more effective, mechanism-based gait rehabilitation interventions for the growing population of older adults at risk for falls and mobility decline.

## 2 Methods

### 2.1 Participants

The data were collected during the human subject experiment previously reported in our study (Khiyara et al., 2025). Here, we analyzed the data of seventeen community-dwelling older adults (6 males/11 females; mean ± standard deviation, age: 73.2 ± 6.0 years; range: 65–92 years; height: 168.7 ± 8.9 cm; mass: 73.1 ± 18.5 kg) who self-reported being right-handed. Participants were required to be able to walk independently for at least 20 min continuously to meet the inclusion criteria. Exclusion criteria included self-reported conditions affecting gait, muscle function, such as peripheral neuropathy, Parkinson’s disease, cerebral palsy, multiple sclerosis, and stroke, reported via an online screening questionnaire. All procedures were approved by the University of Maine Institutional Review Board (IRB 2019-04-15). Written informed consent was obtained from all participants, and data were anonymized to ensure confidentiality.

### 2.2 Experimental protocol

Each participant completed four walking conditions on an indoor standard 200-m track: Baseline, Fast, and two trials with haptic cueing as previously described (Khiyara et al., 2025). The Baseline and Fast conditions were performed without any cueing. In the Baseline condition, participants walked at their self-selected comfortable pace. In the Fast condition, participants walked at their fastest comfortable pace without running. These two walking conditions were conducted on a straight 60-m segment of the track. The Cueing condition utilized a wearable vibrotactile system to deliver bilateral haptic cues, aimed at modulating the timing of arm swing. While there were two Cueing conditions, one to reduce and the other to increase arm swing cycle time (CT), here we focus on the condition to reduce CT by 20%. This condition aimed to increase the frequency of arm swing (i.e., equivalent to shortening the CT) and thereby increase walking speed in the older-adult participants. Participants were instructed to synchronize peak shoulder flexion with the onset of vibration on that arm. The Cueing condition was performed over a full 200-m lap around the indoor track, and the participants were familiarized with the rhythmic cueing on their arms before the experimental trial.

### 2.3 Data collection

**FIGURE 1:**
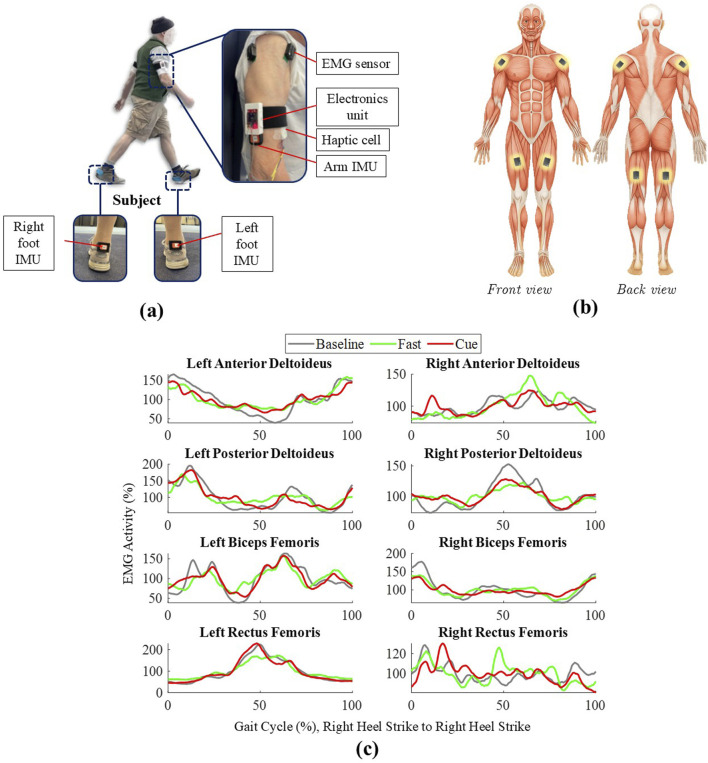
**(a)** Participant with the haptic cueing system, including arm sEMG/IMU sensors, electronics unit, haptic cell, and foot IMUs. **(b)** Schematic of EMG sensor placement on AD, PD, RF, and BF (bilaterally). **(c)** Grand-averaged EMG envelopes over one normalized gait cycle, all segmented by the right heel strike (RHS, 0%–100%) for Baseline, Fast, and Cueing conditions.

As shown in Figure 1a, each participant’s arm was equipped with a haptic cueing unit secured with Velcro straps on the lateral side of each brachium, positioned midway between the shoulder and elbow. The location was chosen because it sits on soft tissue, avoids joints, and allows the device to remain comfortable and stable during walking, consistent with previous work (Noghani et al., 2023; Khiyara et al., 2025). Each haptic electronic unit consisted of an ESP8266 microcontroller, a battery, and a custom circuit board, which was connected to a haptic cell containing three vibrotactors. The haptic cell was oriented toward the front of the arm to deliver cues aligned with the forward motion of arm swing (Figure 1a). The vibrotactors vibrated at 240 Hz, a frequency that falls within the optimal response range of Pacinian mechanoreceptors, making the vibrations easily detectable and producing a clear tactile sensation (Noghani et al., 2021; Noghani et al., 2023; Khiyara et al., 2025; Sharafian et al., 2025). Each vibration cue lasted 100 ms, a duration previously shown to produce a distinct and easily perceived sensation during walking (Noghani et al., 2021; Noghani et al., 2023; Khiyara et al., 2025). An Inertial Measurement Unit (IMU) mounted on each arm recorded arm swing CT and arm range of motion (ROM). Additional IMUs, embedded in custom 3D-printed heel clips, were placed on the participants’ shoes to capture gait events such as heel strikes and toe-offs. Surface Electromyography (sEMG) sensors were placed on eight muscles (Figure 1b): the shoulder muscles [anterior (AD) and posterior deltoid (PD)] and the upper leg muscles [biceps femoris (BF), and rectus femoris (RF)] of both the left and right limbs, following SENIAM guidelines for electrode positioning (Hermens et al., 1999) and Delsys guidelines for sensor placement (Delsys Inc, 2025; D. Inc, 2023). These specific muscles were selected based on a prior work by Weersink et al. (2021a), who conducted intermuscular coherence analysis during gait between bilateral shoulder muscles (AD and PD) and both proximal and distal lower-limb muscles in healthy participants. They reported that the highest coherence values were observed between shoulder muscles and proximal leg muscles, specifically the BF and RF.

The rhythmic haptic cues were controlled by a custom Android application (Noghani et al., 2021; Noghani et al., 2023) that received real-time foot IMU data at 60 Hz and detected heel strikes based on sagittal foot angle trajectories. The application managed vibrotactor activation by calculating a delay D to maintain rhythmic alternation between arms, based on foot CT measured during the Baseline condition (Khiyara et al., 2025). This approach is based on 1:1 frequency coupling between arms and legs during normal speed walking (Hejrati et al., 2017), in which arm and foot CTs were matched (Noghani et al., 2023; Hejrati et al., 2016). Cueing delay D was computed using Equation 1, as defined by Noghani et al. (2023):

$$D=k\times \frac{CT_{f,N}}{2}-t$$ (1)

where $CT_{f,N}$ is the foot CT from the Baseline condition, k is the cue coefficient for Cueing, which was 0.8 for this study, and t is a fixed delay of 100 ms. The target arm CT for the Cueing condition $\left(CT_{c}\right)$ was given by Equation 2 (Noghani et al., 2023):

$$CT_{c}=k\times CT_{f,N}$$ (2)

Coefficients k<1 produced shorter arm CTs, while k≥1 increased them. For example, if $CT_{f,N}=1000$ ms, a k=0.8 would result in $CT_{c}=800$ ms and D=500 ms. After the 10th heel strike detection in each Cueing condition, the Android app transmitted the calculated D to the ESP8266 microcontrollers via HTTP, which then triggered alternating vibration sequences.

sEMG signals were recorded at a sampling rate of 1926 Hz using the Trigno Wireless Biofeedback System by D. Inc. (2023) with the manufacturer’s built-in 20–450 Hz band-pass filter. Although this setting attenuates sub-20 Hz power, coherence remains mathematically preserved when the same linear time-invariant filter is applied to both channels used in analysis; therefore, α (8–15 Hz) and low- β (15–30 Hz) coherence are unaffected by the hardware filter (Chen et al., 2018; van Asseldonk et al., 2014; Chen et al., 2022). This system includes wireless sensors designed for high-fidelity EMG acquisition, ensuring precise capture of muscle activity during gait (D. Inc, 2023). Sensors were placed on cleaned skin surfaces and oriented parallel to the muscle fibers to optimize signal quality. The EMG signals were transmitted wirelessly to a base station and stored on a laptop for subsequent analysis (D. Inc, 2023). Kinematic signals were recorded separately using Xsens IMUs (Xsens Technologies B.V., Enschede, The Netherlands). After data collection, the EMG and IMU recordings were synchronized by cross-correlating their triaxial acceleration signals from the same body segment (upper arm) and shifting the EMG data accordingly. This procedure aligned both systems to a common time base, ensuring consistent gait event timing. Once synchronized, gait events such as heel strikes and toe-offs were identified from the IMU data and used to segment the EMG signals into individual gait cycles.

### 2.4 Spatiotemporal and kinematic data processing

Kinematic parameters, including walking speed, arm ROM, and arm ROM symmetry ratio, were calculated from the IMU data recorded on the arms and feet. Sagittal plane angles of the upper limbs were derived from the arms’ IMUs. Arm ROM was computed as the angular difference between maximum shoulder flexion and extension within a gait cycle. Arm ROM symmetry was calculated as the ratio of right arm ROM divided by left arm ROM, where values greater than 1 indicate a larger right arm ROM, values less than 1 indicate a larger left arm ROM, and values closer to 1 indicate greater symmetry (Khiyara et al., 2025). Walking speed was calculated by integrating linear acceleration data from foot-mounted IMUs using a zero-velocity update algorithm (Dadashi et al., 2013; Hossain et al., 2023). To ensure analysis of steady-state walking, the first 10 gait cycles of each walking condition were discarded, and the subsequent 30 consecutive gait cycles were analyzed (Khiyara et al., 2025; Noghani et al., 2023).

### 2.5 Preprocessing EMG data

The preprocessing for intermuscular coherence and directionality analysis closely followed the process described by Weersink et al. (2021a), with adjustments to account for different experimental conditions and the implementation of algorithms using MATLAB 2024b (MathWorks Inc. 2024). The raw sEMG signals were first high-pass filtered at 5 Hz using a finite impulse response (FIR) filter. The FIR filters were selected for their stability and linear phase response, which preserves the temporal structure of the signal (Litwin, 2000). MATLAB’s designfilt and filtfilt functions were used to implement the filter and ensure zero-phase distortion. This process effectively removed the low-frequency noise, including baseline drift and movement artifacts commonly introduced during gait. Following filtering, the sEMG signals were rectified using full-wave rectification, which converted all negative values to positive (Konrad, 2005; Weersink et al., 2021a). The filtered and rectified signals were segmented into individual gait cycles based on right heel strikes identified from the Xsens IMU data, consistent with prior EMG studies that used right-heel-strike segmentation (Kim et al., 2016; Kwon et al., 2023; Weersink et al., 2021a). The first 10 steps of each condition were excluded to ensure the analysis of steady-state walking, and 30 gait cycles were analyzed for each participant (Sharafian et al., 2025; Khiyara et al., 2025). For consistency, the 30 analyzed gait cycles during the cueing condition were extracted from the same straight 60-m portion of the 200-m track as used in the Baseline and Fast conditions.

The duration of each gait cycle was calculated, and the sEMG data were time-warped using linear interpolation to align each cycle to the individual’s average gait cycle duration. The time-warped sEMG envelopes were normalized by expressing them as a percentage of the mean activity of the individual within each condition (Weersink et al., 2021a). The normalized sEMG envelopes were smoothed using a moving average filter with a 10-ms window to further refine the signal. This preprocessing pipeline, which included time-warping, normalization, and smoothing, was applied separately for each condition, exclusively for visualization purposes, to generate the grand-averaged EMG envelopes shown in Figure 1c. For intermuscular coherence analysis, only the sEMG signals high-pass filtered at 5 Hz and rectified were used (Weersink et al., 2021a; 2022a). The signals were segmented into individual gait cycles based on right heel strikes (Weersink et al., 2021a); no time-warping was applied, and all original gait cycle durations were retained to avoid frequency distortion. Time-warping to 0%–100% of the gait cycle was applied only after the spectral analysis, strictly for coherence heatmap visualization.

### 2.6 Coherence and directionality analysis

Time-dependent intermuscular coherence was computed using a sliding-window Fourier analysis, following the framework of Halliday et al. (1995) as also implemented in more recent gait studies (Weersink et al., 2021a; Weersink et al., 2022a). The sEMG signals from the upper and lower limbs were segmented with a 200 ms window sliding in 50 ms steps across each gait cycle. This produced a series of time offsets (i.e., the window positions) relative to the heel-strike event. For each offset, auto-spectral densities $f_{xx}\left(\omega \right)$ and $f_{yy}\left(\omega \right)$ and the cross-spectral density $f_{yx}\left(\omega \right)$ were estimated by averaging FFT-based periodograms across all gait cycles at that offset. The magnitude-squared coherence between signals x and y at frequency ω was then calculated using Equation 3 (Halliday et al. 1995):

$$|R_{yx}\left(\omega \right){|}^{2}=\frac{{\left|f_{yx}\left(\omega \right)\right|}^{2}}{f_{xx}\left(\omega \right)f_{yy}\left(\omega \right)}$$ (3)

This procedure yields a time-dependent coherence spectrum at each offset, $|R_{yx}\left(\omega \right){|}^{2}$ , characterizing how intermuscular coupling strength varies over the gait cycle and across frequencies. Coherence values were estimated across three frequency bands: alpha (8–15 Hz), beta (15–30 Hz), and gamma (30–60 Hz), which are commonly associated with distinct neural sources. Specifically, alpha-band coherence is associated with subcortical drive, particularly from brainstem structures such as the reticulospinal tract, and is often linked to automatic control of rhythmic movement (Conway et al., 1995). In contrast, beta- and gamma-band coherence are both associated with corticospinal contributions from the sensorimotor cortex (Salenius et al., 1997; Baker and Baker, 2003; Borhanazad et al., 2024), but we analyzed them separately because they are thought to reflect different motor control processes. Beta coherence is typically stronger during steady, continuous movements and is linked to maintaining stable motor output and sustained sensorimotor integration (Baker and Baker, 2003; Borhanazad et al., 2024), whereas gamma coherence tends to appear during rapid or changing movements and may support brief, task-specific bursts of corticospinal drive (Mima et al., 2000; Brown et al., 1998; Borhanazad et al., 2024).

For each participant, coherence was computed individually and later pooled across participants to derive group-level coherence estimates. Coherence estimates were averaged over gait cycles and time offsets within each condition. Following the approach by Halliday et al. (Halliday et al., 1995) and Weersink et al. (Weersink et al., 2021a), significant coherence values (P<0.05) were identified and prepared for visualization in the time-frequency heat maps.

To assess the directionality of coupling, we applied the non-parametric decomposition method (Halliday, 2015), which separates coherence into forward, reverse, and zero-lag components. First, the sEMG signals were pre-whitened to remove autocorrelation structure while preserving their coherence. Each signal’s Fourier transform was divided by its amplitude spectrum (i.e., the square root of its autospectrum), yielding whitened processes with flat unit spectra $f_{xx}^{w}\left(\omega \right)=f_{yy}^{w}\left(\omega \right)=1$ . The cross-spectrum of the whitened signals, $f_{yx}^{w}\left(\omega \right)$ , is therefore equal in magnitude to the original coherence spectrum. We then performed an inverse Fourier transform on $f_{yx}^{w}\left(\omega \right)$ to obtain the time-domain cross-correlation function between the two signals, which is given by Equation 4 (Halliday, 2015):

$$\rho_{yx}\left(\tau \right)=\frac{1}{2\pi }\int_{-\pi }^{\pi }f_{yx}^{w}\left(\omega \right)e^{i\omega \tau }d\omega$$ (4)

The resulting time-domain function, $\rho_{yx}\left(\tau \right)$ , was separated into lag ranges corresponding to reverse-lag (negative lags), zero-lag, and forward-lag (positive lags) interactions. Each segment was individually transformed back into the frequency domain, producing three coherence components, as described in Equation 5 (Halliday, 2015):

$$|R_{yx}\left(\omega \right){|}^{2}=|R_{yx;-}^{\prime }\left(\omega \right){|}^{2}+|R_{yx;0}^{\prime }\left(\omega \right){|}^{2}+|R_{yx;+}^{\prime }\left(\omega \right){|}^{2}$$ (5)

These components reflect direction-specific coherence: forward-lag indicates upper-limb influence on lower limb, reverse-lag indicates lower-limb influence on upper limb, and zero-lag reflects common input. Directional coherence was computed for each participant and condition, and the results were used to quantify task-specific modulation of interlimb neural coupling. All computations were implemented in MATLAB 2024a.

### 2.7 Statistical analysis

Walking speed, arm ROM, and arm ROM ratio were analyzed using linear mixed-effects models in SPSS v29 (IBM Corp., Armonk, NY, USA), similar to the statistical approach used in our previous work (Khiyara et al., 2025). Walking condition (Baseline, Fast, Cueing) was included as a fixed main effect, with participant ID as a random effect to account for repeated measurements within participants. Gender was modeled as a fixed factor, and age and BMI were included as covariates. BMI was calculated from measured height and body mass using the standard formula: body mass (kg) divided by height squared (m^2^). Two-way interaction terms between gender, age, and BMI were included in the models to assess potential moderating effects on gait outcomes. Post hoc pairwise comparisons between walking conditions were performed with Bonferroni-adjusted confidence intervals (CI) to control for multiple comparisons. All statistical analyses were conducted at a significance level of α=0.05 . Grand mean coherence values within each frequency band (alpha: 8–15 Hz, beta: 15–30 Hz, gamma: 30–60 Hz) were compared across walking conditions using non-parametric Friedman’s ANOVA for related samples. When a significant main effect was observed, *post hoc* Wilcoxon signed-rank tests were performed for pairwise comparisons, and p-values were adjusted using the Benjamini–Hochberg false discovery rate (FDR) procedure to control for multiple testing (Benjamini and Hochberg, 1995; Benjamini and Yekutieli, 2001). Statistical comparisons of forward and reverse coherence components were performed using Wilcoxon signed-rank tests on the area under the curve (AUC) within the alpha (8–15 Hz), beta (15–30 Hz), and gamma (30–60 Hz) frequency bands. To account for multiple comparisons and limit false positives, p-values were adjusted using the Benjamini–Hochberg FDR procedure (Benjamini and Hochberg, 1995; Benjamini and Yekutieli, 2001). A significance level of α=0.05 was used for all tests. This statistical approach was based on the methods described by Weersink et al. (2021a).

## 3 Results

### 3.1 Gait speed and arm swing metrics

**FIGURE 2:**
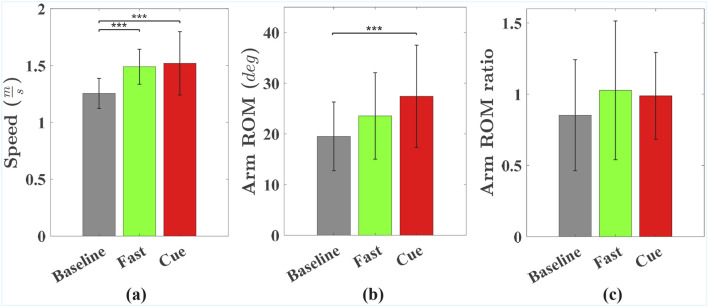
Group means and standard deviations for **(a)** gait speed, **(b)** arm ROM, and **(c)** arm ROM ratio across Baseline, Fast, and Cueing conditions. Significant pairwise differences are indicated by asterisks: * p<0.05 , ** p<0.01 , *** p<0.001 .

Figure 2 shows group means and standard deviations for walking speed, arm ROM, and arm ROM ratio across the Baseline, Fast, and Cueing conditions. The pairwise comparison results are displayed in Figure 2, in the form of asterisks for the p value and a line connecting the significant bar plots. In Figure 2a, a significant main effect of condition was found for gait speed ( F(2,32)=25.33 , p<0.001 , $\eta_{p}^{2}=0.613$ ). Pairwise comparisons revealed that gait speed was significantly higher in both Fast and Cueing compared to Baseline ( p<0.001 for both), with no significant difference between Fast and Cueing. These findings suggest that rhythmic cueing elicited a gait speed increase similar to that observed with self-selected Fast walking.

As shown in Figure 2b, arm ROM was significantly affected by walking condition ( F(2,32)=11.02 , p<0.001 , $\eta_{p}^{2}=0.408$ ). Pairwise comparisons revealed that arm ROM was significantly greater in the Cueing condition compared to Baseline walking (p<0.001) , with no significant differences between Baseline and Fast or between Fast and Cueing. Although not statistically significant, the average arm ROM during the Cueing condition was 16.5% higher than during the Fast condition. This trend suggests that rhythmic haptic cueing can increase arm swing amplitude beyond that achieved during voluntary Fast walking, aligning with previous findings (Khiyara et al., 2025).

Finally, as shown in Figure 2c, there was a trend indicating that walking condition may have affected the arm ROM ratio ( F(2,32)=3.13 , p=0.057 , $\eta_{p}^{2}=0.164$ ), though this effect did not reach statistical significance. An arm ROM ratio value of 1 indicates perfect symmetry between the left and right arms. To assess the degree of symmetry in each condition, one-sample t-tests were performed against a reference ratio of 1. The Baseline condition (0.853±0.390) was not significantly different from 1 (p=0.139) , nor were the Fast ( 1.027±0.487 , p=0.821 ) and Cueing ( 0.989±0.305 , p=0.881 ) conditions. Pairwise comparisons between walking conditions showed no statistically significant differences between Baseline, Fast, and Cueing after correction for multiple comparisons. Although the differences were not statistically significant, these results suggest that trends toward improved arm swing symmetry were observed in Fast and Cueing compared to Baseline. Consistent with our previous findings (Khiyara et al., 2025), some participants exhibited individual improvements in arm symmetry, underscoring the potential for personalized effects of rhythmic cueing even in the absence of statistically significant group-level differences.

### 3.2 Intermuscular coherence

**FIGURE 3:**
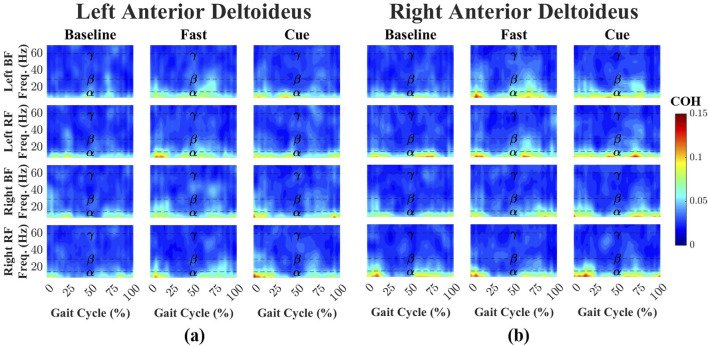
Time–frequency coherence heatmaps between the left **(a)** and right **(b)** AD and bilateral lower-limb muscles (BF and RF) across three walking conditions: Baseline, Fast, and Cueing. Coherence is plotted over the normalized gait cycle based on the right heel strike (x-axis) and frequency range 0–70 Hz (y-axis). Frequency bands are denoted by dashed lines: α (8–15 Hz), β (15–30 Hz), and γ (30–60 Hz). Color indicates coherence magnitude (0–0.15), with warmer colors representing stronger coupling.

**FIGURE 4:**
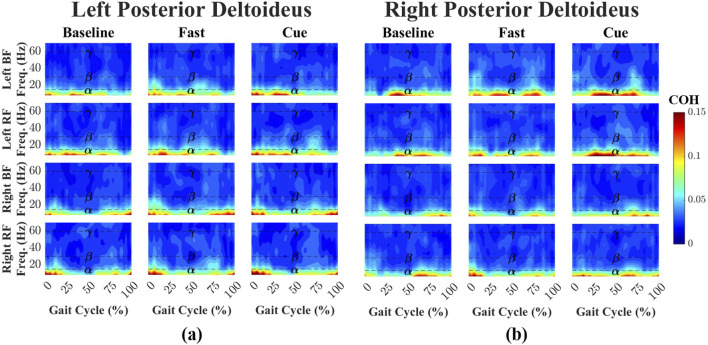
Time–frequency coherence heatmaps between the left **(a)** and right **(b)** PD and bilateral lower-limb muscles across Baseline, Fast, and Cueing conditions. Coherence is plotted over the normalized gait cycle (x-axis) and frequency range 0–70 Hz (y-axis). Frequency bands are denoted by dashed lines: α (8–15 Hz), β (15–30 Hz), and γ (30–60 Hz). Color indicates coherence magnitude (0–0.15), with warmer colors representing stronger coupling.

Time-dependent intermuscular coherence was computed between the anterior deltoid (AD) and posterior deltoid (PD) muscles (left and right), as well as the bilateral biceps femoris (BF) and rectus femoris (RF) muscles. Figures 3, 4 show time–frequency coherence heatmaps for the Baseline, Fast, and Cueing walking conditions. Coherence is plotted across the normalized gait cycle percentage.

For the left AD-leg muscle pairs (Figure 3a), coherence during Baseline walking was primarily confined to the α band (8–15 Hz; mean = 0.0449) with low magnitude. For the β (15–30 Hz; mean = 0.0339) and γ band (30–60 Hz; mean = 0.0291), coherence values were minimal. During the Fast condition, α coherence increased slightly (mean = 0.0547) and extended over a larger portion of the gait cycle, accompanied by a small rise in β coherence (mean = 0.0415) and γ coherence (mean = 0.0331). Cueing further increased α coherence (mean = 0.0571) and produced a modest gain in β coherence compared to Baseline (0.0372 vs. 0.0339), while γ coherence showed only a small change from Baseline (0.0311 vs. 0.0291) and remained low. Pairwise analysis of individual muscle combinations (Supplementary Figures S1–S3) indicated significant α -band increases from Baseline to Fast and from Baseline to Cueing for the Left AD–Right RF pairing (p<0.05) , and a β -band increase from Baseline to Fast for the Left AD–Left RF pairing (p<0.05) . For the right AD-leg muscle pairs (Figure 3b), Baseline α -band coherence was higher (mean = 0.0533) than for the left AD-leg muscle pairs. β (mean = 0.0352) and γ (mean = 0.0325) coherence remained low. In the Fast condition, α coherence increased further (mean = 0.0558), β coherence rose to 0.0411, and γ coherence showed a small increase to 0.0320. Cueing produced the highest α coherence compared to Baseline (0.0610 vs. 0.0533) and Fast (0.0610 vs. 0.0558), and slightly elevated β coherence compared to Baseline (0.0402 vs. 0.0352), with γ coherence remaining low and comparable to Baseline (0.0297 vs. 0.0325). Pairwise results (Supplementary Figures S1–S3) revealed a significant γ -band increase from Baseline to Fast for the Right AD–Left BF pairing (p<0.05) .

For the left PD-leg muscle pairs (Figure 4a), coherence during Baseline walking was dominated by the α band (8–15 Hz; mean = 0.0568) across most pairings; β - (15–30 Hz; mean = 0.0368) and γ -band (30–60 Hz; mean = 0.0279) coherence were minimal. In the Fast condition, α coherence increased (mean = 0.0660) and became more consistently distributed across the gait cycle. This was accompanied by a rise in β coherence (mean = 0.0418) and a slight increase in γ coherence (mean = 0.0297). Cueing further increased α coherence (mean = 0.0684) compared to Baseline (0.0568) and Fast (0.0660), while β coherence slightly decreased from Fast (0.0383 vs. 0.0418) and γ coherence remained low (mean = 0.0292). Pairwise analysis (Supplementary Figures S1–S3) revealed significant α -band increases from Baseline to Fast for the Left PD–Right BF pairing (p<0.01) and from Baseline to Cueing for the same pairing (p<0.05) . For the right PD (Figure 4b), Baseline α -band coherence was strong (mean = 0.0582) similar to the coherence of the left PD. In both the β and γ bands, coherence was low (means = 0.0355 and 0.0290, respectively). In the Fast condition, α coherence increased to 0.0612, β coherence rose to 0.0377, and γ coherence to 0.0305. Cueing produced the highest α coherence compared to Baseline and Fast (0.0665 vs. 0.0582 and 0.0612), while β coherence was similar to Fast and above Baseline (0.0375 vs. 0.0377 and 0.0355), and γ coherence remained low and close to Baseline (0.0291 vs. 0.0290). Coherence was generally higher between the right PD and contralateral leg muscles than with ipsilateral leg muscles. Pairwise results (Supplementary Figures S1–S3) indicated a significant β -band increase from Baseline to Fast for the Right PD–Left BF pairing (p<0.05) .

Across all conditions and frequency bands, PD–leg coherence values were consistently higher than AD–leg coherence, although none of these differences reached statistical significance. This trend was most apparent in the α band, where PD–leg coherence exceeded AD–leg coherence in Baseline (p=0.1588) , Fast (p=0.1676) , and Cue (p=0.2000) conditions. In the β and γ bands, coherence values between AD and PD were similar, with no clear visual separation.

**FIGURE 5:**
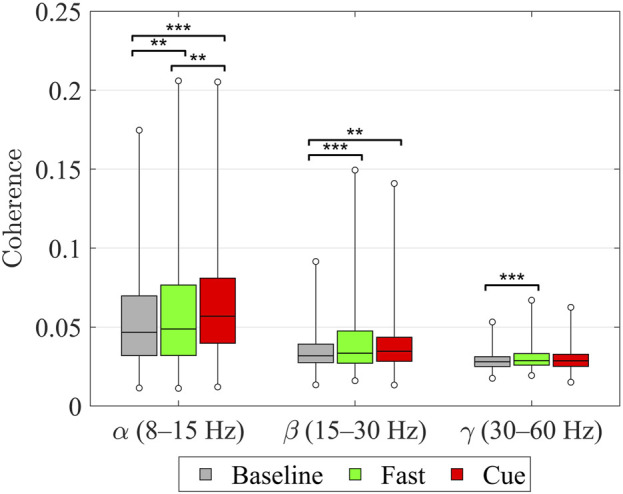
Intermuscular coherence between upper and lower limb muscle pairs within the α (8–15 Hz), β (15–30 Hz), and γ (30–60 Hz) frequency bands across walking conditions (Baseline, Fast, Cueing). Each box represents the median (horizontal line) and interquartile range (IQR), with whiskers indicating the true minimum and maximum values across all subject–pair combinations. Statistical differences were assessed using non-parametric Friedman’s ANOVA for related samples on the subject–pair data, followed by Wilcoxon signed-rank tests with Benjamini–Hochberg FDR correction for multiple comparisons. Significant pairwise differences are indicated by asterisks: * p<0.05 , ** p<0.01 , *** p<0.001 (after FDR correction).

Finally, as shown in Figure 5, when coherence values across all upper–lower limb muscle pairs were analyzed using Friedman’s ANOVA, significant effects of walking condition were found in all three frequency bands. In the α band, coherence was significantly greater during Fast compared to Baseline (p<0.01) , during Cueing compared to Baseline (p<0.001) and during Cueing compared to Fast (p<0.01) . In the β band, both Fast and Cueing conditions exhibited significantly higher coherence than Baseline ( p<0.01 with Cueing and p<0.001 with Fast). In the γ band, coherence was significantly greater during Fast compared to Baseline (p<0.001) . These findings indicate that faster walking and rhythmic haptic cueing are associated with increased intermuscular coherence between the upper and lower limbs, with the largest relative increases observed in the α band.

### 3.3 Directionality coherence

**FIGURE 6:**
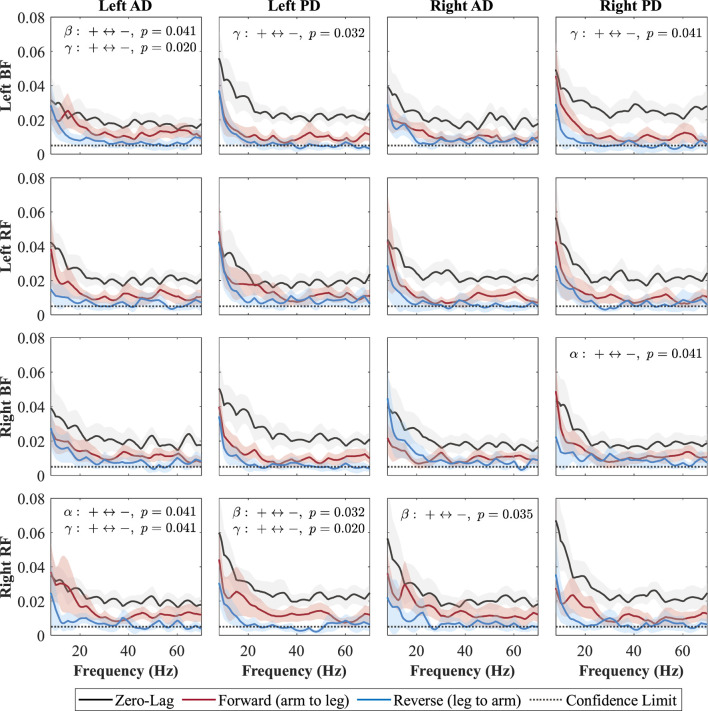
Directional coherence spectra for Baseline between upper- and lower-limb muscles. Subplots (4 × 4) show zero-lag (0, black), forward-lag (+, red), and reverse-lag (–, blue) coherence; shaded areas are 95% CIs, and the horizontal dotted line marks the 95% confidence threshold for significance, assuming the time series are uncorrelated. P-values for AUC comparisons (+vs. –) are annotated by (+ ↔ -) in each subplot.

**FIGURE 7:**
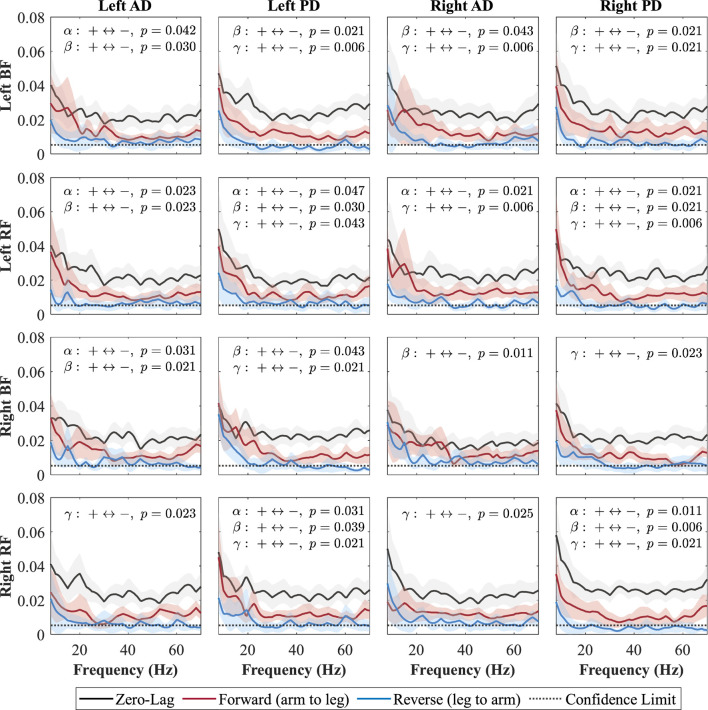
Directional coherence spectra for Fast between upper- and lower-limb muscles. Subplots (4 × 4) show zero-lag (0, black), forward-lag (+, red), and reverse-lag (–, blue) coherence; shaded areas are 95% CIs, and the dotted line marks significance. P-values for AUC comparisons (+vs. –) are annotated by (+ ↔ -) in each subplot.

**FIGURE 8:**
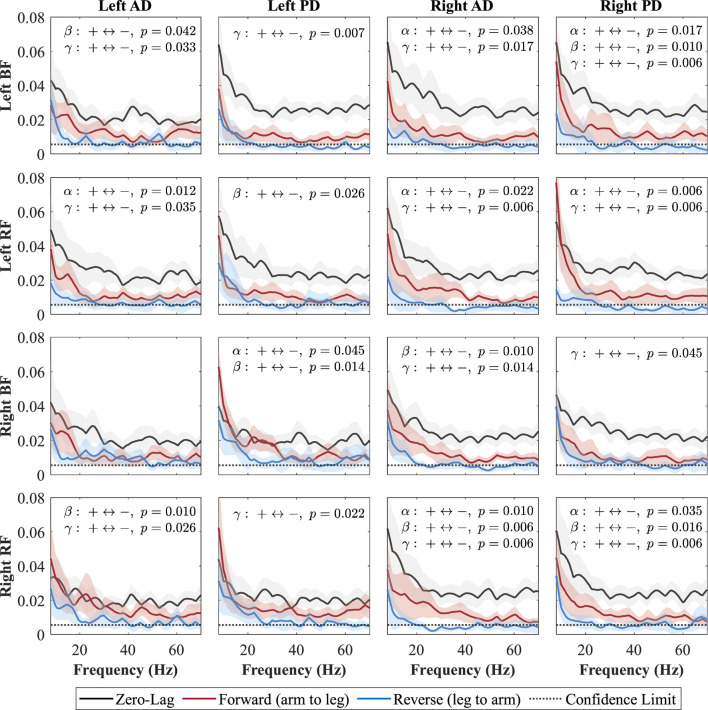
Directional coherence spectra for Cueing between upper- and lower-limb muscles. Subplots (4 × 4) show zero-lag (0, black), forward-lag (+, red), and reverse-lag (–, blue) coherence; shaded areas are 95% CIs, and the dotted line marks significance. P-values for AUC comparisons (+vs. –) are annotated by (+ ↔ -) in each subplot.

To determine the direction of neural influence, coherence was decomposed into forward-lag ( + ; arm → leg), reverse-lag ( − ; leg → arm), and zero-lag (0; shared input) components using Halliday’s non-parametric directionality framework (Halliday, 2015). Figures 6–8 illustrate the directional coherence spectra results between upper- and lower-limb muscles for the Baseline, Fast, and Cueing conditions, respectively, across alpha (8–15 Hz), beta (15–30 Hz), and gamma (30–60 Hz) frequency bands.

Each subplot shows a muscle pair with zero-lag (0, black line; shared neural drive), forward-lag (+, red line; arm-to-leg influence), and reverse-lag (–, blue line; leg-to-arm influence) coherence components. Shaded areas denote 95% CIs and the dotted horizontal line marks significance/confidence limit. Consistent with Weersink et al. (Weersink et al., 2021a), only the FDR-corrected Wilcoxon p -values for the forward versus reverse ( +↔ − ) area-under-the-curve comparison are annotated. We did not perform statistical comparisons involving the zero-lag component, as it reflects shared (common) neural drive rather than directional influence; thus, we focused on comparing forward and reverse components only.

During the Baseline condition (Figure 6), zero-lag coherence was present in every shoulder–leg combination, confirming a strong shared drive. After FDR correction, the forward-lag component exceeded the reverse-lag component in several specific pairs, indicating a top-down arm → leg influence. The left AD–left BF pair showed significant beta- (p=0.041) and gamma-band (p=0.020) coherence. Additional gamma-band shoulder-to-leg coupling was observed for the left PD–left BF (p=0.032) and right PD–left BF (p=0.041) pairs. In the alpha band (8–15 Hz) , a significant effect emerged for right PD–right BF (p=0.041) and for right RF–left AD (p=0.041) ; the latter pair also reached significance in the gamma band (p=0.041) . The right RF–left PD pair exhibited significant beta (p=0.032) and gamma (p=0.020) coherence, while right RF–right AD was significant in the beta band (p=0.035) . Reverse-lag coherence never surpassed forward-lag for any shoulder–leg pair or frequency band.

In the Fast condition (Figure 7), forward-lag coherence consistently exceeded reverse-lag coherence for all shoulder–leg combinations, with significant effects occurring in at least one of the frequency bands (alpha, beta, or gamma) for each pair. In the Cueing condition (Figure 8), a larger number of shoulder–leg pairs reached significance in the forward-versus reverse-lag comparison compared to Baseline, further reflecting a strengthened top-down influence during rhythmic haptic cueing, which was used to synchronize arm swing to a target rhythm designed to increase walking speed and arm ROM. Significant forward-lag coherence was especially prevalent in the gamma band, with multiple ipsilateral and contralateral muscle pairings surpassing the confidence limit and showing statistical significance after correction. As shown in Figure 8, the detailed p -values for all significant pairs indicate that rhythmic haptic cueing increases the number of muscle pairs exhibiting forward dominance and broadens the frequency range of this effect relative to the Baseline condition. One exception is the left AD–right BF pair, which, similar to Baseline, did not reach significance in the Cueing condition; in the Fast condition, this pair exhibited significant forward-lag coherence in both the alpha and beta bands.

For every subject–pair combination, the AUC of the zero-, forward-, and reverse-lag spectra was integrated within each frequency band. Each AUC was then expressed as a percentage of the sum of the three components. These percentages were averaged across participants within a muscle pair and subsequently across all the pairs; the resulting grand means and standard deviations are reported in Table 1.

**TABLE 1:** Grand mean ± SD from per-pair means for Baseline, Fast, and Cueing.

Condition	Frequency band	Zero-lag	Forward-lag	Reverse-lag
Baseline	Alpha	48.4 ± 4.4	29.9 ± 4.4	21.8 ± 5.0
Baseline	Beta	53.2 ± 5.5	28.6 ± 4.2	18.1 ± 4.2
Baseline	Gamma	54.8 ± 4.9	27.5 ± 2.5	17.7 ± 3.7
Fast	Alpha	48.8 ± 5.4	31.6 ± 3.2	19.7 ± 4.7
Fast	Beta	54.3 ± 5.1	29.9 ± 3.1	15.9 ± 3.2
Fast	Gamma	56.5 ± 4.6	27.8 ± 2.1	15.7 ± 3.3
Cueing	Alpha	49.3 ± 6.6	31.6 ± 5.1	19.1 ± 4.3
Cueing	Beta	55.3 ± 6.3	28.5 ± 4.6	16.2 ± 3.7
Cueing	Gamma	58.3 ± 5.3	26.8 ± 2.5	14.9 ± 3.8

Across all conditions, zero-lag coherence accounted for approximately half of the total coherence, indicating that interlimb coupling was primarily driven by a common (zero-lag) input. Forward-lag coherence was consistently greater than reverse-lag coherence, reflecting a predominant arm → leg influence. In each condition, the zero-lag component increased from the alpha to the gamma band, while reverse-lag showed the opposite trend. Zero-lag coherence also showed a small but consistent increase from Baseline to Fast to Cueing across all frequency bands, with reverse-lag decreasing accordingly. These patterns suggest a modest strengthening of the common drive and a reduction in the leg → arm component with faster gait and rhythmic haptic cueing.

**FIGURE 9:**
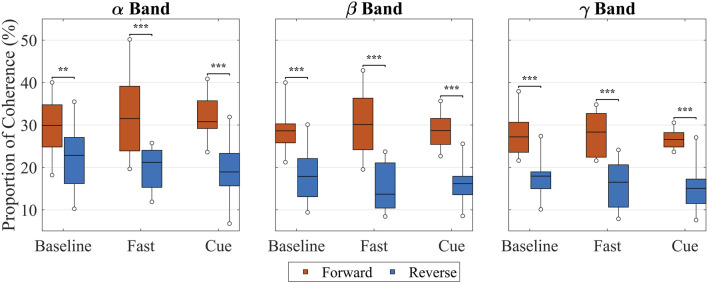
Proportion of forward (red) and reverse (blue) intermuscular coherence in the α -, β -, and γ -bands across walking conditions (Baseline, Fast, Cue). Data represent all muscle pairs combined to provide an overall comparison of upper-to lower-limb coherence direction. Box plots show the median, IQR, and whiskers from the true minimum to the maximum observed values, thereby illustrating variability across all participants. Significance bars indicate results of within-subject comparisons between directions for each condition (** p<0.01 , *** p<0.001 ; paired Wilcoxon tests with Benjamini–Hochberg FDR correction).

Building on the pair-level averages in Table 1; Figure 9 presents box plots including all participants’ data in each frequency across the conditions. For each participant, coherence values were averaged across all shoulder–leg pairs to provide an overall upper-versus lower-limb value in each frequency band and condition. Then, the data of all participants were used to calculate the box plots for each frequency band for each condition. Across all conditions, the separation between forward- and reverse-lag coherence was evident across all frequency bands. In Baseline, forward- and reverse-lag coherence were the closest, differing by less than 10% across all frequency bands. In contrast, both Fast and Cueing showed a larger separation between forward and reverse components in every band, indicating a clearer predominance of arm → leg influence under these conditions. While forward-lag coherence was significantly greater than reverse-lag coherence in all conditions, the effect was particularly strong (p<0.001) in all cases other than the Baseline α -band, where the difference was also significant (p<0.01) . These plots illustrate variability across individuals and confirm that the forward dominance observed in the averages was consistent at the individual level.

## 4 Discussion

### 4.1 Overview of findings

This study demonstrates that rhythmic haptic cueing of arm swing significantly influences gait performance and enhances neuromuscular coordination in older adults. Specifically, the Cueing condition, in which arm swing cycle time was reduced, resulted in a significant increase in arm range of motion (ROM) and a corresponding significant improvement in gait speed compared to Baseline. A similar trend was observed in the Fast condition, where gait speed increased significantly, though increases in arm ROM did not reach significance. Although improvements in arm ROM ratio were not statistically significant at the group level, individual-level enhancements were observed. These behavioral improvements coincided with increased intermuscular coherence in the alpha and beta bands, and a notable presence of gamma-band coherence in directionality analysis in the forward-lag (arm to leg) component. Together, these results suggest that the cueing intervention activated both subcortical and cortical pathways to enhance interlimb coordination.

### 4.2 Interpretation of gait metrics

The significant increase in gait speed observed in the Cueing condition reinforces the hypothesis that modulating arm swing can influence lower-limb output through interlimb coupling, as reported previously (Khiyara et al., 2025). While Fast walking also increased gait speed, the Cueing condition achieved comparable improvements without consciously trying to walk faster, highlighting the potential of externally driven arm swing training for older adults with reduced mobility. The significant 40.6% increase in arm ROM during Cueing compared to Baseline, along with a 16.5% increase relative to Fast (not significant), suggests that rhythmic cueing can elicit greater upper-limb engagement than both normal and voluntary fast walking. Previous studies have highlighted the importance of arm swing amplitude in enhancing trunk stability and reducing energy expenditure during gait (Ortega et al., 2008; Meyns et al., 2013). Our findings are consistent with this body of research, supporting the view that increased arm swing amplitude contributes to both biomechanical and neuromuscular efficiency (Huang and Ferris, 2004; Umberger, 2008). Although changes in arm ROM ratio did not reach statistical significance at the group level (p=0.057) , inspection of individual data revealed that several participants moved closer to perfect symmetry in the Cueing condition. This suggests the potential of subject-specific responsiveness to rhythmic cueing, highlighting the need for further research into personalized cueing protocols.

### 4.3 Intermuscular coherence

Intermuscular coherence is a neurophysiological measure that quantifies the correlation in the frequency domain between EMG signals recorded from two distinct muscles, reflecting the common neural input shared between their motor unit pools (Dos Santos et al., 2020; De Freitas et al., 2025; Weersink, 2021). This shared neural input may originate from cortical, subcortical, or spinal pathways that collectively coordinate muscle activation during rhythmic tasks such as walking (Farmer et al., 1993; Grosse et al., 2004; Weersink et al., 2021a; De Freitas et al., 2025). Intermuscular coherence is typically stronger between muscle pairs with close anatomical and functional relationships, making it a valuable tool for exploring neural circuitry involved in motor control and detecting impairments in these pathways (Farina et al., 2004).

The present coherence results indicate that both normal walking (Baseline and Fast) and rhythmic haptic cueing engage multiple levels of the central nervous system. At the group level (Figure 5), walking condition significantly modulated intermuscular coherence between upper and lower limbs in all bands: in the alpha-band, coherence increased stepwise (Fast > Baseline; Cue > Baseline; Cue > Fast); in the beta band, both Fast and Cue exceeded Baseline; and in the gamma band, Fast exceeded Baseline whereas Cue did not. These effects were quantified with Friedman’s ANOVA on the full set of subject–pair values, followed by Wilcoxon signed-rank tests with Benjamini–Hochberg FDR correction. The increase in alpha coherence with Fast, and most prominently with Cueing, is consistent with strengthened common subcortical drive and spinal patterning, likely mediated by reticulospinal pathways. This is supported by central pattern generators (CPGs) and propriospinal interlimb connections, which form long descending and ascending pathways linking cervical (arm) and lumbar (leg) CPG networks to coordinate diagonal arm–leg movement during gait (Weersink et al., 2021a; Zehr and Duysens, 2004; Dietz, 2003; Klarner and Zehr, 2018). In practical terms, rhythmic haptic cueing can help synchronize the timing of arm and leg movements and modulate CPG output, and our results show that this could amplify the shared neural drive between upper and lower limbs more effectively than simply walking faster, as shown by the significantly higher alpha-band coherence in the Cueing condition (Figure 5). Past work has shown that CPG activity can be tuned by sensory input, including proprioceptive and cutaneous feedback (Dietz, 2003; Zehr and Duysens, 2004; Klarner and Zehr, 2018). In the present study, rhythmic vibration to the arm likely provided cutaneous afferents directly and proprioceptive afferents indirectly through larger and more regular arm swings, both of which could influence CPG output. Consistent with prior work, sensory-driven signals can modulate CPG activity via propriospinal pathways, adjusting rhythmic arm–leg coordination to meet task demands (Dietz, 2003; Zehr and Duysens, 2004; Klarner and Zehr, 2018). Although CPG activity was not measured directly here, the band-specific intermuscular coherence patterns and established neuroanatomy support this interpretation. By enhancing the timing and amplitude of arm movements, cueing may have strengthened the sensory drive from the upper limbs to spinal circuits coordinating gait, which could help explain the greater alpha-band intermuscular coherence often associated with shared subcortical/spinal drive.

The beta-band increases observed in both Fast and Cue compared to Baseline are consistent with previous findings that more challenging or coordinated walking tasks increase EMG–EMG coherence. These beta-band effects likely reflect added corticospinal contributions for sensorimotor integration and adaptive control under higher demand and tighter timing constraints (De Freitas et al., 2025; Da Silva Costa et al., 2024; Kerkman et al., 2020). Similarly, Kerkman et al. (2020) reported increased intermuscular coherence in the alpha and lower beta bands during 1:1 arm–leg coordination. In our study, Fast walking likely promoted this natural 1:1 coupling, as typically seen at higher speeds (Tester et al., 2012), whereas the Cueing system enforced it by delivering rhythmic arm vibrations in synchrony with the contralateral heel strike (Noghani et al., 2021; Noghani et al., 2023; Khiyara et al., 2025). This precise synchronization likely strengthened the interlimb coupling that contributed to the observed increases in alpha- and low beta-band coherence.

The significant, though visually small, gamma increase with Fast (but not Cue) suggests that high-frequency neural coupling is more influenced by the effort and focus needed for faster walking than by the timing effects of external cueing. Gamma-band coherence is typically observed during rapid or changing movements and is thought to reflect brief, task-specific bursts of corticospinal drive (Mima et al., 2000; Brown et al., 1998; Borhanazad et al., 2024). The greater gamma coherence in the Fast condition likely reflects the additional corticospinal engagement required for rapid, voluntary gait adjustments. For older adults, this heightened neural demand may have contributed to the greater physical and cognitive effort of walking faster without cueing, whereas rhythmic cueing may have reduced reliance on such high-frequency drive by shifting control toward alpha-band mechanisms, thereby lowering the perceived difficulty. This interpretation aligns with prior work showing that greater task difficulty is associated with increased higher-band coherence (Da Silva Costa et al., 2024; De Freitas et al., 2025).

Overall, these band-specific changes suggest a distributed control framework in which spinal and brainstem circuits (including CPGs and reticulospinal pathways) provide rhythmic patterning and interlimb coupling, while corticospinal contributions scale with the demands of speed and external timing, consistent with established evidence of arm–leg coupling during gait (Weersink et al., 2021a; Kerkman et al., 2020; De Freitas et al., 2025).

When looking at specific muscle pairs (Supplementary Figures S1–S3), significant changes clustered in contralateral (diagonal) shoulder–leg combinations: Left AD–Right RF ( α : Fast > Baseline, Cue > Baseline; p<0.05 ), Right AD–Left BF ( γ : Fast > Baseline; p<0.05 ), Left PD–Right BF ( α : Fast > Baseline, p<0.01 ; Cue > Baseline, p<0.05 ), and Right PD–Left BF ( β : Fast > Baseline; p<0.05 ). Only one ipsilateral effect was observed: Left AD–Left RF ( β : Fast > Baseline; p<0.05 ). This contralateral predominance (i.e., 4 contralateral cases out of 5 significant cases) is consistent with diagonal interlimb coupling in human gait and with proposed propriospinal cervical–lumbar linkages and reticulospinal drive coordinating arm–leg rhythms (Weersink et al., 2021a; Zehr and Duysens, 2004; Dietz, 2003). Notably, the two significant effects involving cueing occurred in the alpha band (Left AD–Right RF, Cue > Baseline, p<0.05 ; Left PD–Right BF, Cue > Baseline, p<0.05 ), suggesting that rhythmic haptic cueing appears to preferentially augment subcortical/spinal shared drive across diagonals. Speed-related effects were also present in the alpha band (Left AD–Right RF, Fast > Baseline, p<0.05 ; Left PD–Right BF, Fast > Baseline, p<0.01 ), but in addition extended into the beta- and gamma bands (Right PD–Left BF, β : Fast > Baseline, p<0.05 ; Left AD–Left RF, β : Fast > Baseline, p<0.05 ; Right AD–Left BF, γ : Fast > Baseline, p<0.05 ), consistent with greater corticospinal engagement under higher demand.

Across all conditions, the posterior deltoid (PD) muscle showed a consistent (but non-significant) trend toward higher alpha-band coherence with proximal leg muscles compared to the anterior deltoid (AD) muscle. This contrasts with findings from Weersink et al. (2021a); Weersink (2021), who showed comparable coherence values between AD and PD with proximal leg muscles in older adults, suggesting a similar level of neural coupling. Our results instead suggest a preferential coupling between the PD and leg muscles, particularly in the alpha-band range, which may reflect greater reliance on subcortical pathways. Given the biomechanical role in shoulder extension during the backward swing phase of gait and its function as a postural stabilizer (Pontzer et al., 2009; Barthelemy and Nielsen, 2010; La Scaleia et al., 2014), the stronger coherence with leg muscles may reflect enhanced interlimb coordination via reticulospinal pathways. This supports the interpretation of our results that the PD contributes more substantially than the AD to rhythmic interlimb coordination during steady-state walking. Given that the present AD–PD contrasts did not reach significance ( α : p=0.1588 –0.2000), this interpretation should be considered as hypothesis-generating, pending future work with greater power and targeted comparisons (PD–leg vs. AD–leg). In the β and γ bands, AD and PD coherence appeared similar, consistent with the lack of clear visual separation.

### 4.4 Directional coherence

The decomposition of coherence into directional components revealed distinct differences in neural influence between the Baseline, Fast, and Cueing conditions. A prominent zero-lag component was consistently observed across all shoulder–leg muscle pairs, suggesting a shared presynaptic drive likely originating from both subcortical sources (e.g., reticulospinal pathways) and cortical contributions (Weersink et al., 2021a). This pattern supports the role of common neural input, possibly stemming from the CPGs, reticulospinal pathways, and cortical (corticospinal) contributions, in coordinating rhythmic upper and lower-limb activity during gait. Because this zero-lag component reflects shared rather than directional influence, no statistical comparisons were performed on it, and the analysis instead focused on the directional forward- and reverse-lag components, following the approach of Weersink et al. (Weersink et al., 2021a).

During the Baseline condition (Figure 6), forward-lag coherence (arm → leg) exceeded reverse-lag coherence (leg → arm) in a limited number of shoulder–leg muscle pairs, with significant effects emerging primarily in the beta and gamma frequency bands. These findings indicate a modest top-down influence from arm to leg muscles during steady-state gait, consistent with previous research (Weersink et al., 2021a), which reported forward-directed coherence in specific muscle combinations, particularly involving the right deltoid, and attributed this pattern to both subcortical and cortical contributions.

Both increasing walking speed and applying rhythmic vibration cueing enhanced forward-lag coherence (arm → leg) compared to Baseline, indicating a stronger top-down influence from the upper to lower limbs. In the Fast condition, forward-lag coherence was greater than reverse-lag coherence in all shoulder–leg muscle pairs, with at least one frequency band ( α , β , or γ ) reaching significance for each pair. This suggests that faster walking increases the influence of arm swing on leg muscles, likely because higher gait speeds demand more precise step timing and foot clearance, requiring stronger phase-specific cortical control (Petersen et al., 2012). While previous lower-limb studies have found limited or inconsistent effects of speed on within-limb synchronization (Halliday et al., 2003; Charalambous and Hadjipapas, 2022), our findings indicate that upper–lower limb directionality is more sensitive to speed, possibly because interlimb coordination needs to increase when gait becomes faster.

In the Cueing condition, forward-lag coherence was also greater than reverse-lag coherence for nearly all muscle pairs, with only one pair (left AD–right BF) not reaching significance. Compared to Baseline, Cueing increased the number of significant pairs and produced effects in the alpha, beta, and gamma band. This pattern likely reflects the combined contributions of bilateral subcortical drive, providing a shared rhythmic signal between arms and legs, and phase-specific cortical adjustments that fine-tune muscle activation during the gait cycle (Charalambous and Hadjipapas, 2022; Weersink et al., 2021a; Grosse and Brown, 2003; Conway et al., 1995; Salenius et al., 1997; Fisher et al., 2012). The vibration-based rhythm may help synchronize arm swing more precisely, reinforcing the shared rhythmic drive while enhancing the accuracy of swing-phase.

Across both Fast and Cueing conditions, forward-lag coherence increased in the alpha, beta, and gamma bands, indicating the parallel involvement of complementary descending pathways. This pattern suggests that increasing walking speed or applying rhythmic haptic cueing strengthens top-down arm-to-leg drive by simultaneously enhancing subcortical pathways (alpha-band coherence) and cortical pathways (beta-/gamma-band coherence). The alpha-band coherence is associated with bilateral, automatic drive from corticoreticulospinal and reticulospinal projections (Charalambous and Hadjipapas, 2022; Weersink et al., 2021a) and may also reflect coordinated output from spinal CPGs that link cervical and lumbar locomotor networks via propriospinal connections (Zehr and Duysens, 2004; Dietz, 2003; Klarner and Zehr, 2018). Beta-/gamma-band coherence reflects corticospinal contributions for phase-specific, goal-directed control (Petersen et al., 2012; Fisher et al., 2012; Lemon, 2008). These pathways work together during walking, with alpha-band and CPG-related drives providing a bilateral framework for rhythmic coordination, and beta/gamma-band activity refining movement precision. Weersink et al. (2021a) reported this coexistence of alpha and beta coherence during normal walking. In our study, this pattern was present at Baseline but was amplified in the Fast and Cueing conditions, with greater magnitude and broader spatial distribution, suggesting that increasing walking speed or providing rhythmic arm swing stimulation can enhance both the shared rhythmic drive and the precise cortical modulation that together coordinate upper–lower limb interactions.

Reverse-lag coherence did not statistically exceed forward-lag coherence in any pair or frequency band in either condition. This asymmetry supports the interpretation of a predominantly descending neural influence from the arm to leg muscles. Weersink et al. (2021a) similarly found no cases where reverse-lag coherence significantly exceeded forward-lag coherence and reported comparable forward and reverse coherence in some muscle combinations during normal walking—consistent with our Baseline results. Our results build on these findings by showing that speed and externally modulated arm swing increase forward-directed coherence. These results reinforce the notion that upper-limb motion is not merely a passive component of gait but can actively drive lower-limb activity through distributed neural circuits involving both the brainstem and motor cortex (Weersink et al., 2021a).

### 4.5 Relationship between gait metrics and intermuscular coherence

To place these neural effects in behavioral context, cueing elicited the largest increase in arm swing, 40.6% relative to Baseline and an additional 16.5% relative to Fast, and the largest increase in alpha-band intermuscular coherence, consistent with stronger sensory inputs that modulates spinal locomotor circuits (Dietz, 2003; Zehr and Duysens, 2004; Weersink et al., 2021a). Both Fast and Cueing conditions increased arm range of motion, which paralleled increases in forward-lag coherence, indicating a stronger top-down influence from arms to legs. Both conditions also increased gait speed and beta-band coherence, whereas only the Fast condition increased gamma-band coherence, suggesting that high-frequency coupling relates more to the effort of voluntary speeding than to speed alone (De Freitas et al., 2025; Da Silva Costa et al., 2024; Kerkman et al., 2020). Overall, cueing may alter the mechanism by which speed is achieved, favoring subcortical/spinal common drive (higher α ) and enhancing directional arm-to-leg influence, rather than broadly upscaling higher-frequency coupling. While these findings highlight potential parallel changes in movement kinematics and neural coupling, we did not examine across-participant correlations between behavioral changes and intermuscular coherence, as the study was not explicitly designed or powered for such analyses; future work with larger, dedicated designs should address these associations.

### 4.6 Clinical and rehabilitation implications

The directional coherence findings carry important implications for gait rehabilitation in older adults and individuals with neurological disorders such as Parkinson’s disease. Cueing that reduces arm swing CT can enhance interlimb neural coupling and increase gait speed without requiring conscious control of walking speed, making it a promising intervention for individuals with fatigue or impaired motor control. Previous studies have shown that passive or externally guided arm swing can improve gait initiation and coordination in individuals with Parkinsonian gait (Weersink et al., 2020; Weersink et al., 2021b; Weersink et al., 2022a; Weersink et al., 2022b; Kawashima et al., 2008). Moreover, the observed increases in beta- and gamma-band coherence suggest that rhythmic cueing may engage cortical pathways and promote neuroplasticity. These findings raise the possibility that long-term cueing interventions could yield lasting improvements in motor control, an area that warrants further investigation in future research.

This dual engagement of subcortical and cortical pathways has important implications for rehabilitation strategies. Rhythmic cueing interventions may be effective precisely because they engage multiple levels of the motor hierarchy simultaneously, potentially facilitating both immediate motor adjustments and longer-term motor learning. In clinical populations with impaired cortical function, the preservation of subcortical responses to rhythmic cues may provide an alternative pathway for motor rehabilitation.

### 4.7 Limitations and future directions

Despite the promising findings, several limitations should be acknowledged. The sample size (N = 17) could limit statistical power, especially for detecting subtle effects in arm swing symmetry. The participants only underwent a short period of walking, completing one lap around the track (200 m) while receiving haptic stimuli during the Cueing condition. In the present study, we focused on upper and proximal lower limb muscles (AD, PD, BF, RF), consistent with prior work by Weersink et al. (2021a), Weersink et al. (2022b), which reported the strongest coherence in these upper–lower limb muscle pairs. This selection of proximal lower-limb muscles was further based on studies employing arm–thigh kinematic coupling to investigate interlimb coordination during gait (Hejrati et al., 2017; Carpinella et al., 2010). However, future work should also examine distal lower-limb muscles, such as the medial gastrocnemius (MG) and tibialis anterior (TA), to provide a more comprehensive view of gait control. Moreover, while EMG analysis offers indirect evidence of neural coupling, it cannot localize specific neural generators. Integrating EEG and EMG could provide greater specificity in identifying cortical contributions. Additionally, this study focused on the acute effects of rhythmic cueing; future work should evaluate the long-term effects of such cueing on gait parameters, neuromuscular coordination, and fall risk. Exploring individual differences in responsiveness to cueing may also support the development of adaptive, personalized gait training systems.

It is worth noting that the distinction between subcortical and cortical contributions based solely on frequency bands should be interpreted with caution. While alpha-band activity is predominantly subcortical, and beta/gamma activity is predominantly cortical, there is likely considerable overlap and interaction between these systems that may not be fully captured by frequency-domain analysis alone.

## 5 Conclusion

Rhythmic haptic cueing that targets arm swing cycle time enhances arm range of motion, gait speed, and interlimb neural coupling in older adults. These behavioral and neural improvements support the incorporation of upper limb-focused cueing systems into gait rehabilitation protocols. While our previous study (Khiyara et al., 2025) demonstrated the benefit of arm swing training in older adults in terms of key spatiotemporal gait parameters, the current study investigated the underlying neural mechanism to explain those improvements. The observed increase in alpha- and beta-band coherence and forward-lag directional influence highlights the active role of the arms in driving coordinated lower-limb activity, advancing our understanding of interlimb neural dynamics during walking. Collectively, these results support the hypothesis that rhythmic arm movement actively contributes to gait coordination through coupled subcortical and corticospinal mechanisms. Targeting this interlimb coupling through rhythmic arm cueing offers a promising rehabilitation strategy for improving gait performance and stability, particularly in older adults and individuals with gait impairments.

## Data Availability

The raw data supporting the conclusions of this article will be made available by the authors, without undue reservation.
