# Supplementary material for: Modulating arm swing via haptic cueing alters interlimb neural coupling in older adults
Source: Front Physiol. 2025 Sep 22;16:1657092. doi: 10.3389/fphys.2025.1657092 (PMC12497753; doi:10.3389/fphys.2025.1657092)
Supplement: Supplementary file 1 [file DataSheet1.pdf]

Supplementary Material

0.1 Figures

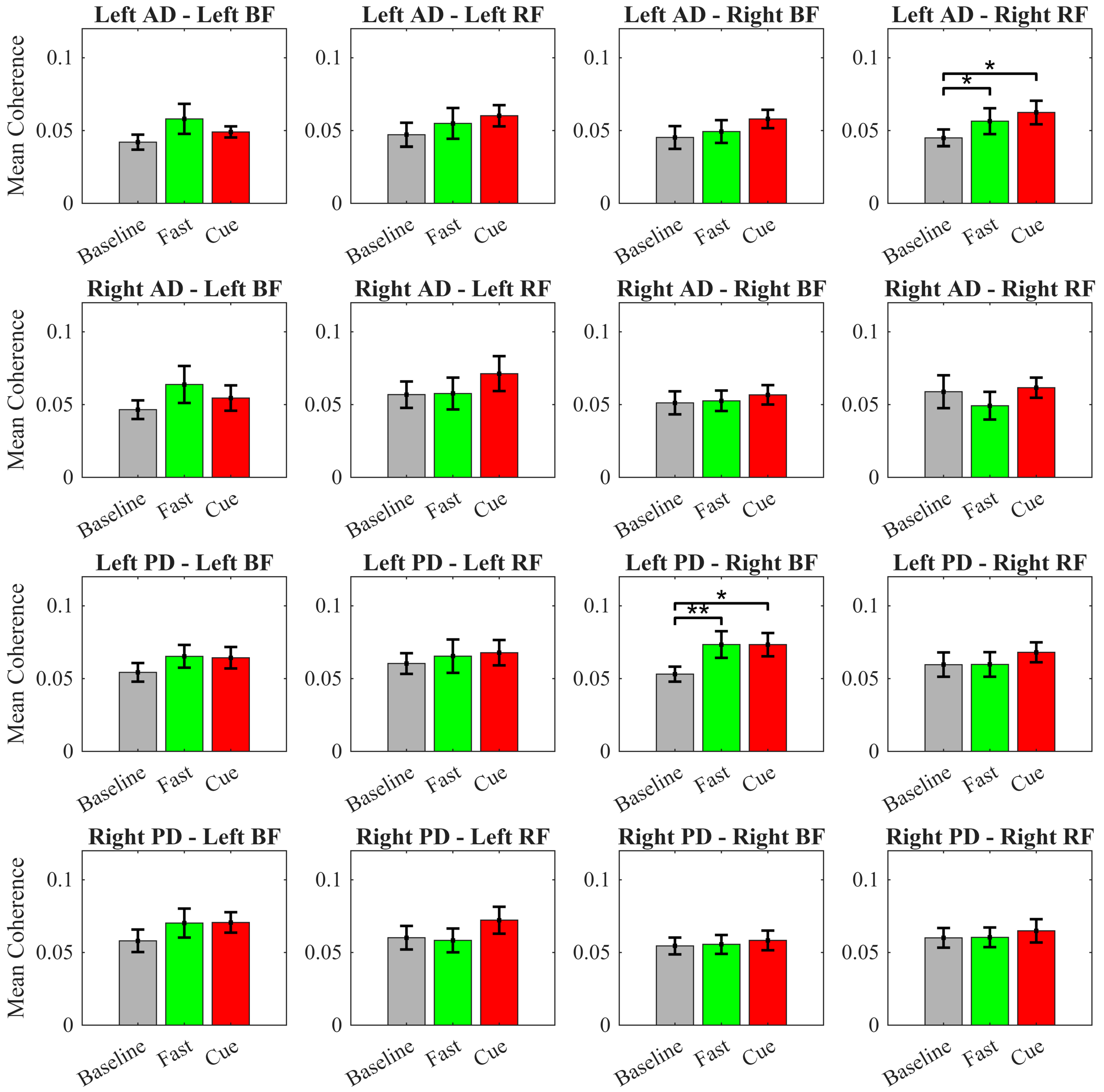

Figure S1. Per-pair mean intermuscular coherence ± SD in the alpha band for all shoulder–leg combinations (Left/Right AD, PD to Left/Right BF, RF). Bars: Baseline (grey), Fast (green), Cueing (red). Asterisks indicate significant differences between conditions (*$p < 0.05$, **$p < 0.01$, ***$p < 0.001$).

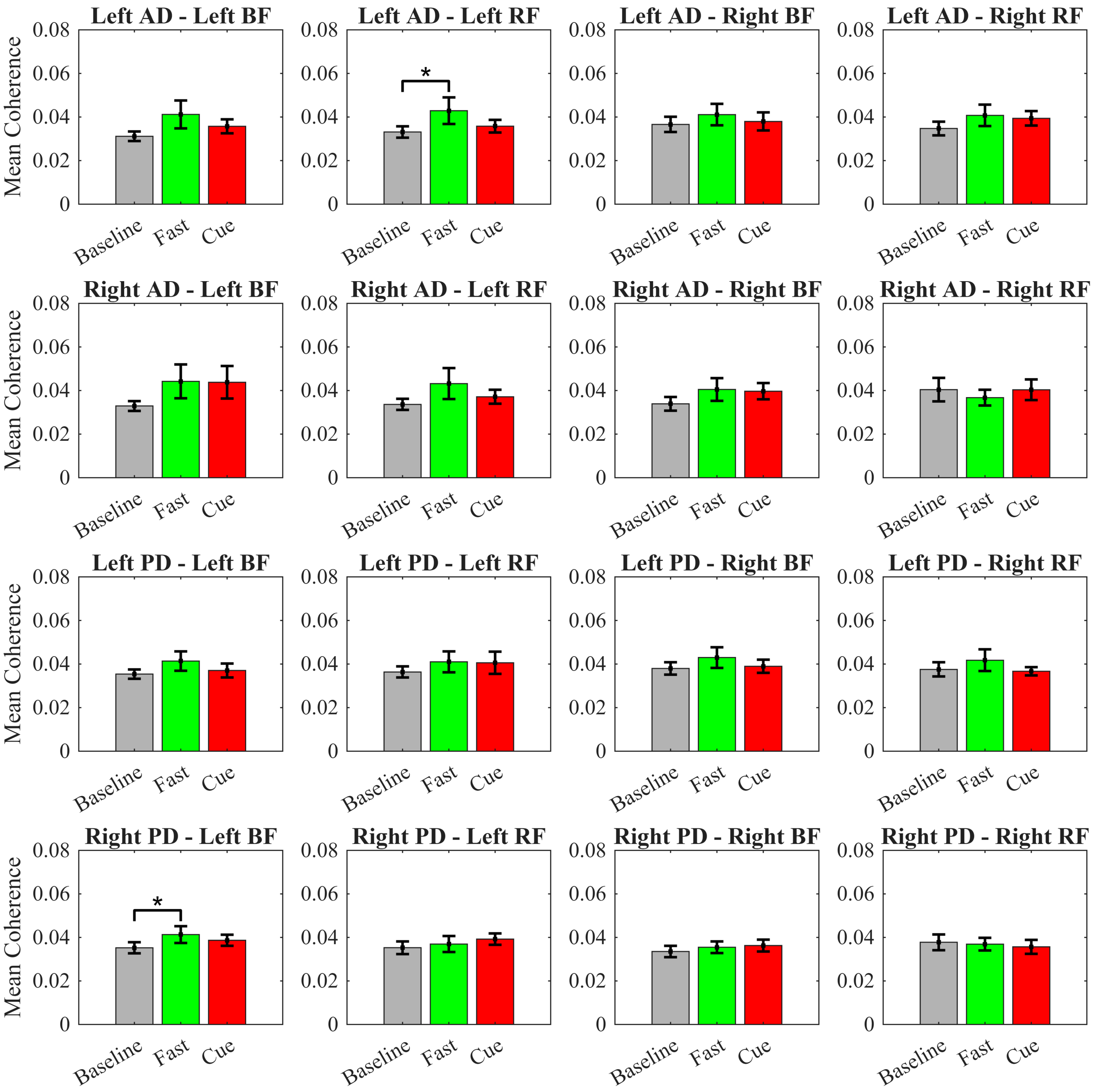

Figure S2. Per-pair mean intermuscular coherence ± SD in the beta band for all shoulder–leg combinations. Bars: Baseline (grey), Fast (green), Cueing (red). Asterisks indicate significant differences between conditions ($*p < 0.05$, $**p < 0.01$, $***p < 0.001$).

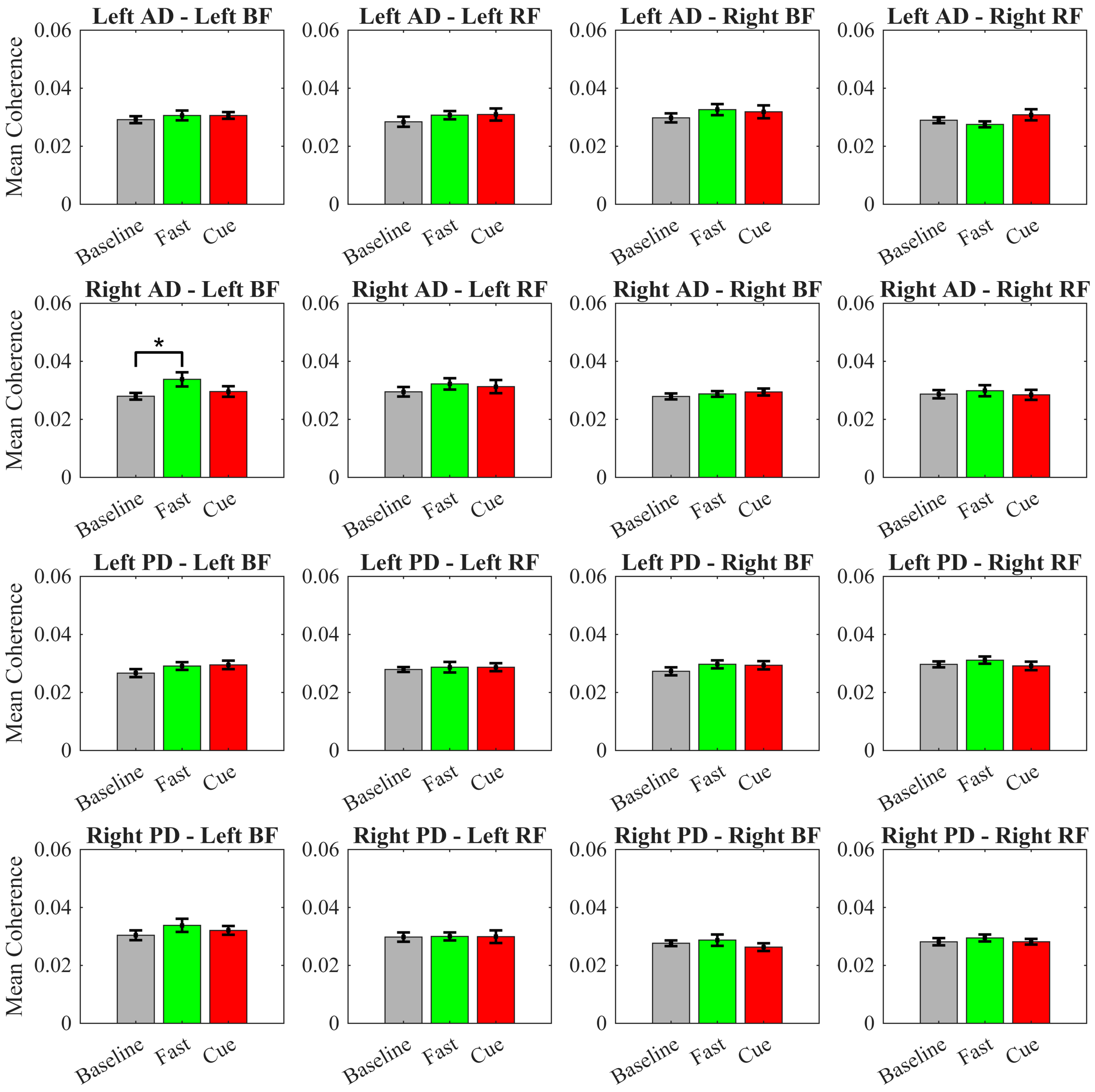

Figure S3. Per-pair mean intermuscular coherence ± SD in the gamma band for all shoulder–leg combinations. Bars: Baseline (grey), Fast (green), Cueing (red). Asterisks indicate significant differences between conditions ($*p < 0.05$, $**p < 0.01$, $***p < 0.001$).
